# Ultrastructural Study of Microphysiological Systems of the Tumor Microenvironment

**DOI:** 10.1002/smsc.202500567

**Published:** 2026-04-24

**Authors:** Paula Guerrero‐López, Karinna Georgiana Pele, Mariano Barrado, Pilar Alamán‐Díez, José Manuel García‐Aznar, Elena García‐Gareta

**Affiliations:** ^1^ Multiscale in Mechanical & Biological Engineering Research Group Aragon Institute of Engineering Research (I3A) School of Engineering & Architecture University of Zaragoza Zaragoza Aragon Spain; ^2^ Advanced Microscopy Laboratory LMA‐University of Zaragoza Zaragoza Aragon Spain; ^3^ Aragon Institute for Health Research (IIS Aragon) Miguel Servet University Hospital Zaragoza Aragon Spain; ^4^ Division of Biomaterials & Tissue Engineering UCL Eastman Dental Institute University College London London UK

**Keywords:** cancer‐on‐a‐chip, extracellular vesicles, microphysiological systems, spheroids, ultrastructure

## Abstract

The importance of studying the ultrastructure is underwritten by decades of research. Ultrastructural features help to understand pathological processes or inform disease diagnosis. However, ultrastructural studies in the promising cancer‐on‐a‐chip models are practically nonexistent because of the complexity of sample preparation for electron microscopy techniques, which is particularly aggravated with these miniaturized models. Our aim was to study ultrastructural features of microphysiological systems (MPS) of the tumor microenvironment consisting of 3D multicellular tumor structures that were grown in hydrogel‐based cancer‐on‐a‐chip models. To this end, we selected two different MPS from our lab as examples and devised a sample preparation technique for their observation under dual‐beam focused ion beam scanning electron microscopy (FIB‐SEM) and transmission electron microscopy (TEM). The proposed methodology allowed high‐resolution visualization of both external and internal organization of 3D multicellular tumor structures, including cell–matrix interactions, cell–cell junctions, spheroid–spheroid contacts, matrix deposition, and extracellular vesicle‐mediated intercellular communication. This study demonstrates the feasibility of using advanced electron microscopy techniques to observe ultrastructural features of miniaturized cancer models, thus revealing a new dimension in the use of these models to study tumor processes and find new therapeutic targets.

## Graphical abstract

We studied ultrastructural features of microphysiological systems (MPS) of the tumour microenvironment consisting of 3D multicellular tumour structures that were grown in hydrogel‐based cancer‐on‐a‐chip models. We selected two different MPS from our lab as examples and devised a sample preparation technique for their observation under dual‐beam focused ion beam scanning electron microscopy (FIB‐SEM) and transmission electron microscopy (TEM). We visualized both external and internal organization of 3D multicellular tumour structures, including cell–matrix interactions, cell–cell junctions, spheroid–spheroid contacts, matrix deposition, and extracellular vesicle‐mediated intercellular communication.

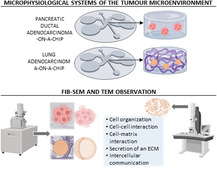

## 1 Introduction

Cancer is a devastating disease and one of the primary causes of death worldwide. Although significant progress has been made in combating it, particularly in terms of early diagnostic and tailored treatments, much research is still needed to understand its causes, development, and spread. Particular focus of this research is on the tumor microenvironment (TME), a unique biochemical, biophysical, and cellular milieu, which plays a crucial role in various aspects of cancer development, progression, and response to therapy [1, 2, 3, 4]. The TME is responsible for regulating tumor cell proliferation, angiogenesis, invasion, and metastasis, while also influencing drug resistance, immune evasion, and the overall aggressiveness of the tumor [2, 4].

Tumor cells in vitro have the ability to assemble into 3D structures that display increasing complexity as well as pathophysiological relevance when provided with suitable stimulation. These multicellular structures include spheroids, tumoroids, and aggregates, either single or multicell type, assembled from tumor cell lines or patient‐derived tumor cells [5, 6, 7]. These 3D structures have gained much attention as physiologically relevant tools to assess response to therapies [5, 8], as they closely mimic the main features seen in avascular solid tumors, which include structural organization as well as oxygen, pH, and nutrients gradients [9].

Microphysiological systems (MPS) encompassing miniaturized hydrogel‐based cancer‐on‐a‐chip models have emerged as powerful platforms to recreate the TME and understand its complexity [10, 11]. These models incorporate tumor cells 3D structures, and present important advantages over other in vitro cancer models, such as biomimicry, minimum use of materials and reagents, control of the physiologically relevant 3D microenvironment, versatility and straightforward monitoring of cell growth and activity using optical, fluorescence, and confocal microscopy, as well as biochemical assays [5, 6, 12, 13, 14, 15]. However, advanced electron microscopy techniques are generally not explored in these MPS due to technical challenges associated with sample preparation and processing. This hinders the observation of key ultrastructural features of the TME, such as cell–matrix interaction, cell–cell structural unions, cell secretion of matrix components, or intercellular communication. While optical and fluorescence‐based methods provide essential molecular and functional information, they cannot resolve many ultrastructural features that define cellular organization at the nanometer scale. Access to such information is critical for comprehensive structural characterization of complex 3D tumor models and a better understanding of the TME.

Research has shown that studying ultrastructural features is crucial for comprehending cellular processes, disease diagnosis, or extracellular matrix (ECM) characterization. The investigation of ultrastructural cell changes can provide insights into cell death, stress responses, hormonal changes, disease mechanisms, or can help distinguish different types of diseases [16, 17, 18, 19]. Therefore, the development of robust and compatible preparation workflows is necessary to enable the application of advanced electron microscopy techniques to MPS. Establishing such methodologies represents a key step towards integrating ultrastructural analysis into the study of engineered TMEs.

The current range of electron microscopy techniques offers versatility and choice of one technique over other for a particular purpose. Scanning electron microscopy (SEM) is the most popular technique offering excellent surface characterization up to the nanoscale detail. Internal structures can also be indirectly accessed through specific sample preparation methods, such as cryo‐fracturing or resin embedding and sectioning. By contrast, focused ion beam SEM (FIB‐SEM) enables direct and sequential milling of the sample, thereby revealing internal ultrastructural features in a more controlled and precise manner [20]. FIB‐SEM would be particularly useful for observing internal ultrastructural features of tumor cells 3D structures. Furthermore, the FIB can be used to prepare thin lamellas for transmission electron microscopy (TEM) observation, further revealing ultrastructural features of tumor cells 3D structures.

The aim of this work was to establish and validate a robust workflow for ultrastructural analysis of MPS of the TME, consisting of 3D multicellular tumor structures grown in hydrogel‐based cancer‐on‐a‐chip models. Two representative MPS developed in our laboratory were selected as case studies to demonstrate the applicability and reproducibility of the proposed methodology. To achieve our aim, we devised a sample preparation technique for observation of miniaturized hydrogel‐based cancer‐on‐a‐chip models under dual‐beam FIB‐SEM and TEM. This study focuses on expanding the methodological toolkit available for the structural investigation of engineered tumor models. By integrating cancer‐on‐a‐chip technology with advanced electron microscopy, we provide a framework that facilitates nanoscale characterization of complex in vitro tumor systems and supports future biological and mechanistic studies.

## 2 Materials and Methods

### 2.1 Study Design

**FIGURE 1:**
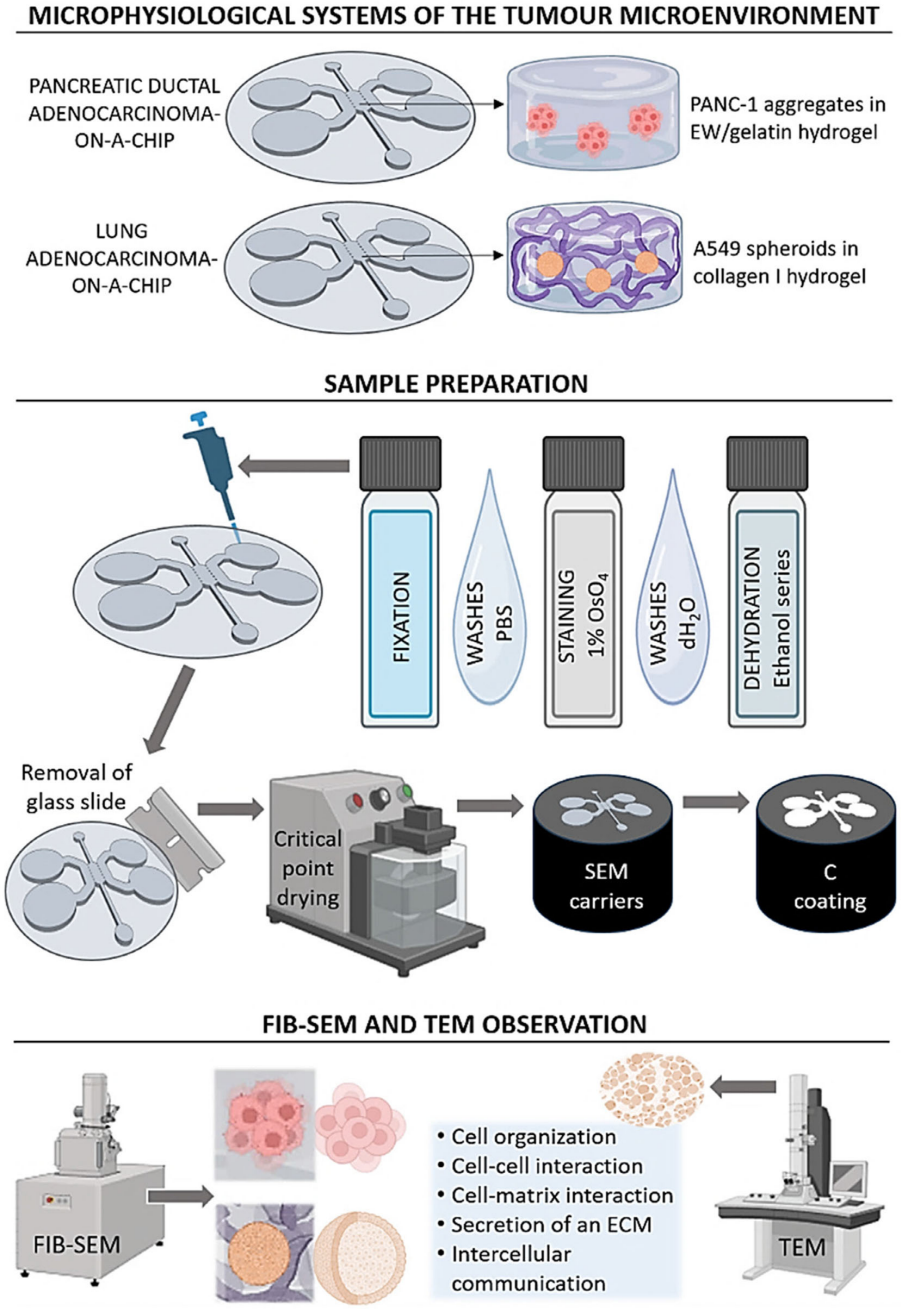
Schematic overview of the study workflow. The MPS consisted of one‐chamber microfluidic devices used to generate two cancer‐on‐a‐chip models: PDAC and LAC. Cells were embedded in natural hydrogels composed of egg white (EW)‐gelatin and collagen type I, respectively. At the end of the culture period, devices were fixed, stained, dehydrated, disassembled, critically point‐dried, mounted, and coated for SEM and FIB‐SEM imaging. For LAC models, the FIB was additionally used to prepare thin lamellae for TEM. This workflow enabled high‐resolution observation of both external and internal organization of 3D multicellular structures, including cell–cell and cell–matrix interactions, matrix deposition, and intercellular communication.

Figure 1 offers a visual schematic of the whole study design, where we used two different MPS from our lab as examples [6, 21]. Our MPS utilized one‐chamber microfluidic devices (fabrication details in Section 2.3) to produce two different cancer‐on‐a‐chip models of the TME, both consisting of 3D multicellular structures embedded in hydrogel‐based matrices. One of the models represented pancreatic ductal adenocarcinoma (PDAC) [6] and the other one lung adenocarcinoma (LAC) [21], through the use of cell lines from these malignancies (PANC‐1 and A549, respectively). Hydrogels used were made of natural materials, namely egg white (EW), gelatin, and collagen type I. Details of cell lines and hydrogels are seen in Figure 1 and described in Sections 2.2 and 2.4. At the end of the culture period, the devices were fixed, stained, dehydrated, disassembled, critically point‐dried, mounted on carriers, and coated with a conducting material for observation under FIB‐SEM (details in Section 2.7). Samples were also milled using the focused ion beam of FIB‐SEM to observe the internal zone of the 3D multicellular structures. Finally, for the models of LAC only, the FIB was used to produce a thin lamella for TEM observation.

Ultrastructural aspects to study from the array of FIB‐SEM and TEM images were cell organization in the 3D multicellular structures, both external and internal, cell–cell interactions, cell–matrix interaction, secretion of ECM, and intercellular communication through extracellular vesicles (EVs). Quantitative analysis was performed where technically and conceptually appropriate, namely quantification of secreted ECM fiber features and EVs. These parameters were selected because they can be reliably identified and measured in grayscale EM datasets with sufficient contrast and boundary definition. A minimum of six microfluidic devices per model were processed and observed.

### 2.2 Cell Culture

Human PDAC cell line PANC‐1, and human LAC cell line A549 from the American Type Culture Collection (ATCC, USA) were used for this work. Cells were cultured in Dulbecco's Modified Eagle Medium (DMEM, Gibco, Spain) with high glucose concentration and supplemented with 10% fetal bovine serum (FBS, Life Technologies, Spain), 100 U/ml penicillin, 100 μg/ml streptomycin, and 2 mM L‐glutamine (all from Lonza, Switzerland). Cultures were maintained in a humidified incubator set at 37°C and 5% CO_2_ until 80% confluence was reached for use in the experiments.

### 2.3 Fabrication of Microfluidic Devices

The device geometry used for the cancer‐on‐a‐chip models consisted of a single central chamber (2.5 × 1.3 mm) containing an array of trapezoidal posts to cage the hydrogels and cells and two parallel side channels as medium reservoirs, all with a height of 300 μm [6, 13, 21]. For microfluidic device fabrication, a silicon wafer (Sandford University) with positive SU8 240‐µm relief patterns obtained by soft lithography was used. Polydimethylsiloxane (PDMS, Sylargd 184, Dow Corning GmbH, Wiesbaden, Germany) was made at a 10:1 weight ratio of base to curing agent. The solution was poured into the SU8 wafer and degassed. The resulting layer was trimmed, perforated and autoclaved. Finally, the PDMS devices were plasma‐bonded to 35 mm glass‐bottom petri dishes (Ibidi, Gräfelfing, Germany).

### 2.4 Fabrication of Hydrogels

In this study, two hydrogels were used to prepare the cancer‐on‐a‐chip models used for ultrastructural observation: EW/gelatin and collagen type I hydrogels. The fabrication and characterization of these hydrogels have been previously detailed [6, 13, 22], but we offer here a brief summary of the fabrication process.

EW/gelatin hydrogels were prepared by cracking open chicken (*Gallus gallus domesticus*) eggs from a local supermarket, isolating the EW and lyophilizing it to obtain a powder that was mixed with gelatin (Type B, from bovine skin, Merck, Spain) in distilled water (dH_2_O) to prepare 5% EW colloidal solutions with 1%–5% gelatin. To mimic physiological pH and prevent cytotoxicity, the pH of the hydrocolloids was adjusted to neutral (pH = 7.0 ± 0.5). These hydrocolloids were used for producing hydrogels by heating at 80°C for 30 min followed by cooling at 4°C overnight.

Hydrogels with a final collagen concentration of 6 mg/ml were prepared by diluting collagen type I solution (Rat Tail, stock 10.8 mg/ml, Corning, Spain), in DMEM (4.5 g/L glucose, Thermo Fisher Scientific, Spain), 10x Dulbecco's phosphate buffered saline (DPBS), and 0.5 M NaOH (both from Sigma‐Aldrich, Germany) to adjust the pH to 7.4–7.6. The mixture was prepared on ice.

### 2.5 Poly‐D‐Lysine Coating

Microfluidic devices to be loaded with collagen hydrogel were coated with poly‐D‐lysine (PDL) to enhance collagen adhesion to the device's surface [13]. A 1 mg/ml solution of PDL hydrobromide (Sigma‐Aldrich, Spain) in cell culture water was made, introduced through the small ports to cover the central chamber and incubated at 37°C for 4 h. The devices were washed with dH_2_O and dried at 80°C overnight.

### 2.6 Cell Seeding and Culture in Microfluidic Devices

EW/gelatin hydrocolloids were made as explained in 2.4 subsection and introduced through the small ports of the microfluidic device. After gelation, 4 µl of PANC‐1 cell suspension (3 × 10^6^ cells/ml) were introduced through the small ports into the already formed hydrogels, then microfluidic devices were kept at 37°C with 5% CO_2_ for 20 min to allow cell attachment. Finally, cell culture media was added through medium channels and the devices kept at 37°C with 5% CO_2_ for 14 days with media changes every 2–3 days.

**FIGURE 2:**
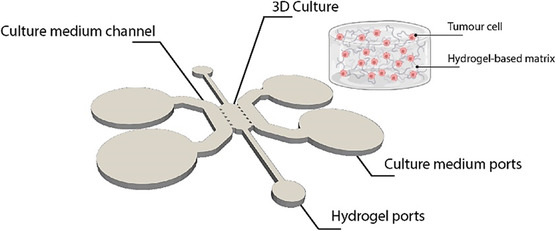
Schematic of a generic MPS used in this study. The inner geometry consists of a single microfluidic channel with ports for hydrogel loading and perfusion of culture medium. The central chamber contains the 3D multicellular tumor culture embedded in a hydrogel matrix. Two distinct cancer‐on‐a‐chip models were implemented, each in a separate device: pancreatic ductal adenocarcinoma (PDAC, PANC‐1 cells) and lung adenocarcinoma (LAC, A549 cells). This schematic highlights the overall architecture of the chip and the spatial arrangement of the tumor constructs, hydrogel, and medium channels.

The collagen I hydrogels at 6 mg/ml were made as detailed previously including the A549 cells within the media (0.2 × 10^6^ cells/ml). The collagen/cells solution was pipetted through the small ports of the microfluidic device and left at 37°C for 20 min to allow gelation. Cell culture media was then added through the medium reservoirs. Devices were then kept at 37°C with 5% CO_2_ for 10 days with media changes every 2–3 days. Figure 2 schematically shows the inner geometry of a cultured MPS prior to preparation for electronic microscopy.

### 2.7 Sample Preparation

Hydrogels inside the microfluidic devices were first fixed with 2% glutaraldehyde in 0.1 M phosphate buffer (pH 7.4) for 1–2 h (LAC‐on‐a‐chip) or 4% paraformaldehyde for 20–30 min (PDAC‐on‐a‐chip) at room temperature, followed by three washes with PBS for 5 min each. A second series of PBS washes (3 × 5 min) was performed to ensure complete removal of glutaraldehyde. Postfixation was carried out with 1% osmium tetroxide (OsO_4_) in phosphate buffer for 1 h in the dark at room temperature. Samples were then washed three times with distilled water (5 min each) and dehydrated through a graded ethanol series as follows: 30% (5 min), 50% (5 min), 70% (5 min × 2), 96% (10 min × 2), and 100% (10 min × 2). Care was taken to ensure complete filling of the central chamber during fixation, staining and dehydration steps. After dehydration, the glass slide from the device was carefully removed by inserting a blade along each edge, starting from the larger inlet ports. Critical point drying (CPD) was performed using the longest available program. Dried samples were mounted onto SEM carriers using conductive double‐sided carbon tape. To enhance their electrical conductivity, a ∼15 nm thin carbon (*C*) film was subsequently deposited onto the specimens via evaporation using a Leica EM ACE200 coater. Please note that for LAC‐on‐a‐chip samples, the collagen I gel was easily detached from the device and therefore, the gel would be mounted on the SEM carrier instead of the whole device. In the sample preparation panel of Figure 1 we chose to just represent the entire device on the SEM carrier for simplicity and clarity.

### 2.8 Dual‐Beam Focused Ion Beam Scanning Electron Microscopy (FIB‐SEM)

To analyze the surface and internal structure of the sample and obtain 3D information, a Cryogenic Dual Beam‐NOVA 200 system was used. To analyze the surface of the sample, accelerating voltages ranging from 200 V to 30 kV were used. To select the targeted area to mill, a high electron voltage (30 KV) was used to visualize internal features and accurately locate the region of interest. Once identified, a protective platinum (Pt) layer was deposited on the surface by first electron beam and finally ion beam, to reduce damage during the cross sections milling. The FIB was then used to precisely cut into the sample, allowing us to monitor the process through in situ SEM imaging. Finally, the cross‐section was polished using a lower ion beam current to achieve high‐resolution SEM images of the target region. In our case, Pt layer was deposited in RT but the rest of process was made in cryo conditions, about −100°C, to minimize damage sample. At the beginning, we used medium‐low currents (0.3 nA–50 pA) for milling and, to do the fine polishing, we used low currents (50–10 pA).

### 2.9 Transmission Electron Microscopy (TEM)

To prepare lamellas for high‐resolution TEM analysis, a rectangular area was selected and milled from both the front and back sides of the Pt protective layer. Given the softness of our material, reduced ion beam currents (0.5–0.3 nA) were used to minimize damage. Once the prelamella reached a thickness of approximately 1–1.5 µm, it was cut until nearly detachment from the bulk. A micromanipulator (Omniprobe) was then employed to extract the lamella and place it onto a TEM grid. Finally, the lamella was thinned by stepwise polishing with progressively lower ion beam currents until it reached a thickness of approximately 100 nm, suitable for TEM imaging. To further analyze the morphology and structure of the lamella, TEM was performed in a Tecnai T20 microscope (ThermoFisher, formerly FEI) at a working voltage of 200 KV. TEM images were acquired with a Veleta 2K × 2K CCD camera.

### 2.10 Extracellular Matrix Fibers Measurements

The diameter and length of fibers secreted by PANC‐1 cells was measured from FIB‐SEM surface images using Fiji ImageJ software. Four images were used, from which 98 measurements were made for fiber diameter and 46 measurements were made for fiber length.

### 2.11 Extracellular Vesicles (EVs) Size Measurement

The size of EVs was measured from FIB‐SEM section images using Fiji ImageJ software. For PANC‐1 cells in EW/gelatin hydrogel five images were used, from which 48 measurements were made in total. For A549 cells in collagen hydrogel four images were used, from which 59 measurements were made in total.

### 2.12 Data Analysis

Data were analyzed descriptively using GraphPad Prism 8. Measurements of matrix fibers and EVs are presented as violin plots and histograms to illustrate distributions and variability within each condition. In addition, Figure S1 presents a visual guide describing the image analysis approach used to evaluate the acquired images.

## 3 Results

### 3.1 MPS of the TME of Pancreatic Ductal Adenocarcinoma: PANC‐1 Cells in Egg White/Gelatin Hydrogels

**FIGURE 3:**
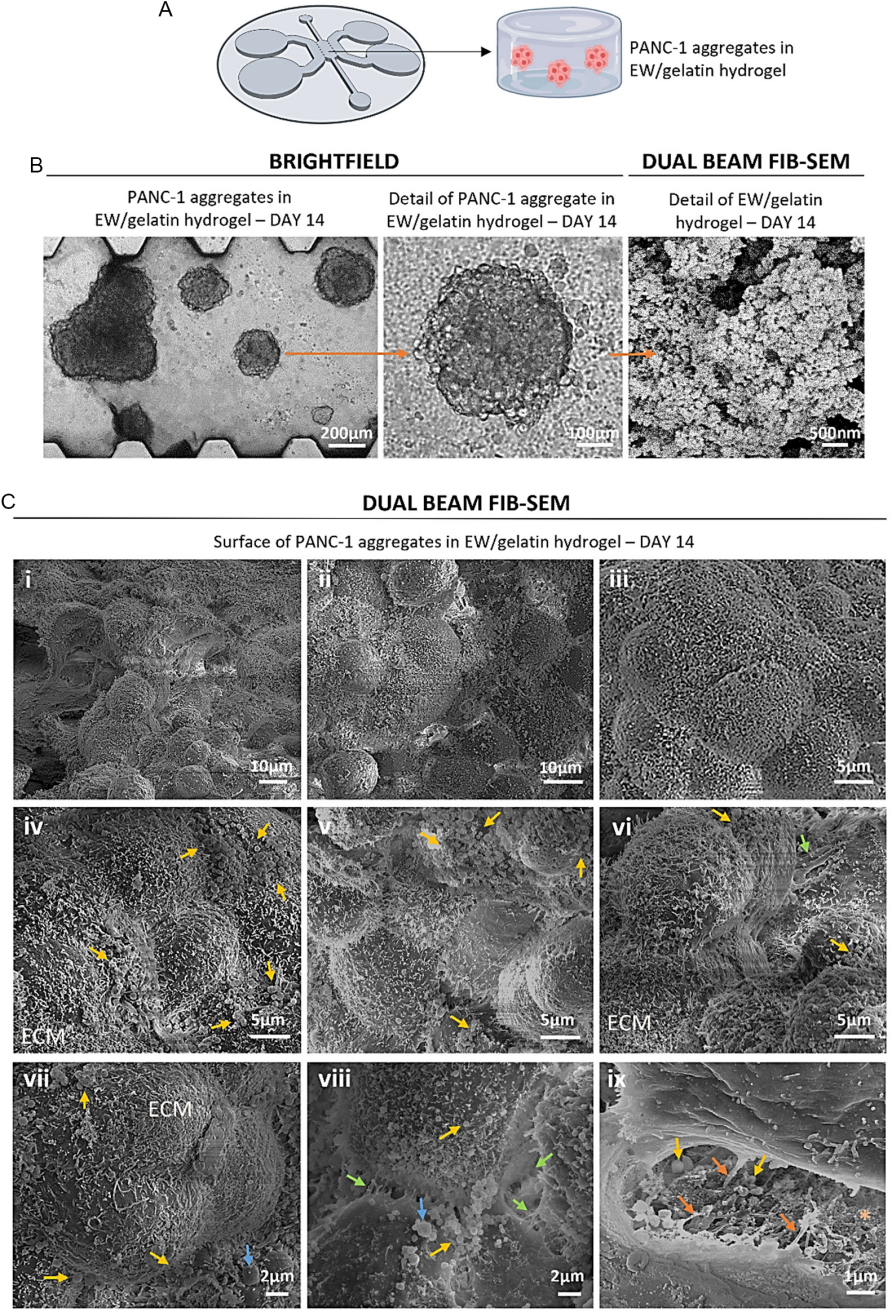
(A) Scheme of the MPS of the TME of PDAC, consisting of a hydrogel‐based cancer‐on‐a‐chip model where the PANC‐1 cell line was used. (B) Brightfield microscopy representative images of the PANC‐1 aggregates grown over 14 days of culture in EW/gelatin hydrogels. Zoomed area shows single aggregate. Dual beam FIB‐SEM representative image of EW/gelatin hydrogel after 14 days of culture, displaying the typical nanoglobular morphology of these hydrogels. (C) Dual beam FIB‐SEM representative images of the surface of PANC‐1 aggregates in EW/gelatin hydrogel. ECM: extracellular matrix produced by the cells. Orange asterisk: EW/gelatin hydrogel. Green arrows: unions between cells. Orange arrows: unions between the cells and the hydrogel. Yellow arrows: spherical particles. Blue arrows: large spherical particles.

The EW/gelatin hydrogels used to produce the PDAC models were developed and thoroughly characterized by our group in previous studies [6, 22], highlighting the unique nanoglobular architecture of these gels as well as their tunable viscoelastic properties. When cells from the human PDAC cell line PANC‐1 were cultured in these EW/gelatin hydrogels they formed large aggregates that resembled grape‐like clusters (Figure 3A,B), which in a previous study by our group we attributed to the unique nanoglobular morphology (Figure 3B) as well as the rheological properties of the EW/gelatin hydrogel [6]. In this previous study, we quantified the size of these aggregates, which could be up to 0.45 mm^2^ (Figure S2), and we also briefly showed some FIB‐SEM representative images of the morphology of these structures, acknowledging that further research was necessary to thoroughly characterize their morphological features as well as their interaction with the environment [6]. Here, we observed these aggregates in minute detail (Figure 3C).

**FIGURE 4:**
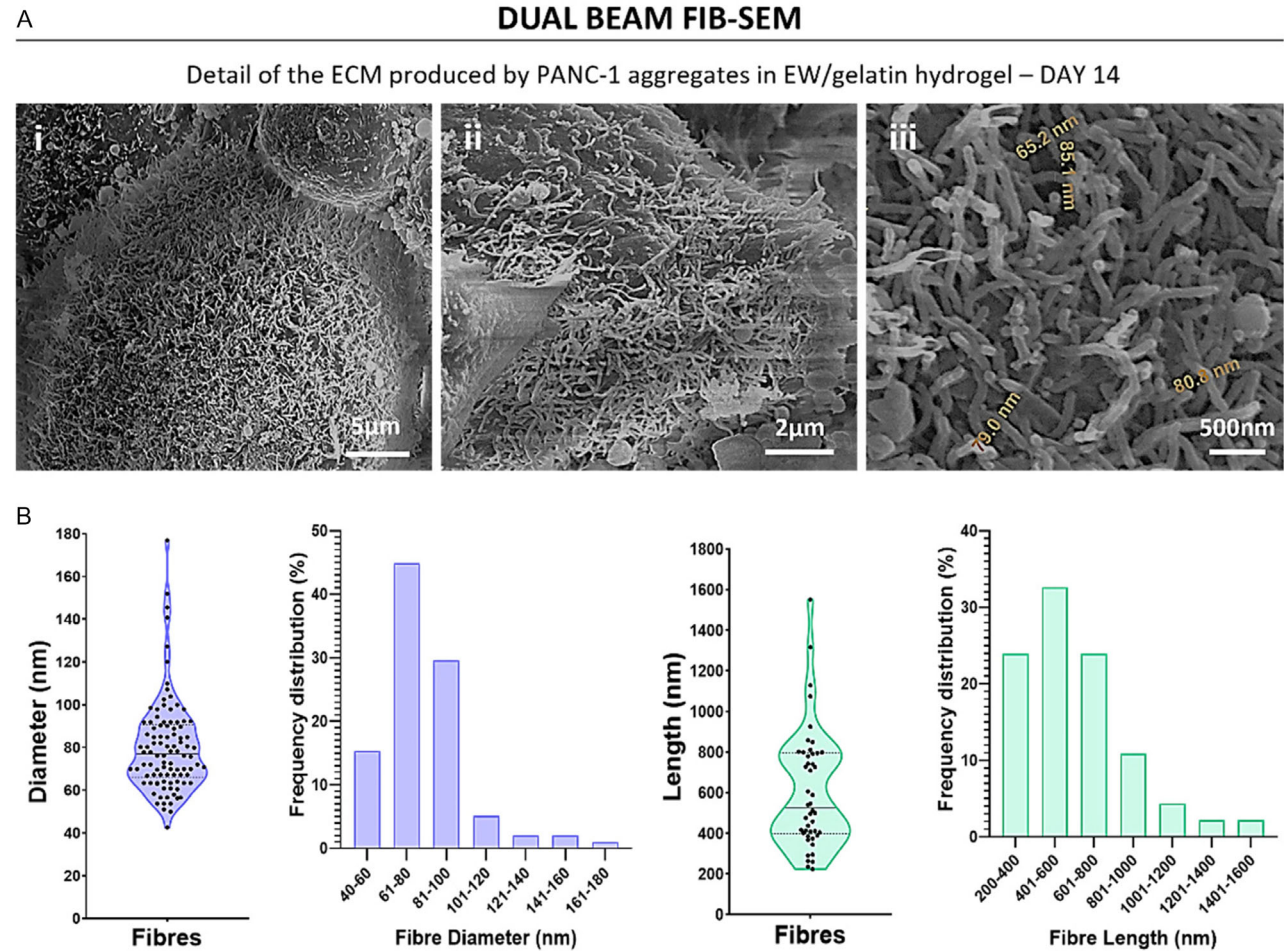
(A) Dual beam FIB‐SEM representative images of the ECM secreted by the PANC‐1 cells. Image iii shows some measurements of the diameter of the fibers that comprise the ECM. (B) Measurements of the diameter and length of the ECM fibers secreted by the PANC‐1 cells. Data shown in violin plot as its distribution with median and the interquartile range (IQR), each dot represent a measurement (*n *= 98 for fiber diameter and *n* = 46 for fiber length).

In terms of fine detail of external cell organization as observed under FIB‐SEM, we saw that the PANC‐1 aggregates had indeed a grape‐like cluster appearance, and the individual cells could be discerned (Figure 3C). The aggregates appeared compact and exhibited granular surfaces of stacked cells, which were 5–10 μm in diameter and presented rounded morphology. A structured dense matrix distributed over the cells surface that was secreted by the cells was observed: see as examples ECM in Figure 3C‐iv,vi,vii. This matrix was seen to form a dense coating over the cells (Figure 4A‐i,ii) and was formed by short fibers (Figure 4A‐iii) that seemed to have random orientation. Measurements of the diameter and length of these fibers showed that the diameter was between 40 and 180 nm in size, with the majority of fibers in the 50–100 nm range (Figure 4B). The length of these fibers was between 200 and 1,600 nm, the majority of which were found in the 200–800 nm range (Figure 4B).

Microvilli were seen on the cell surface embedded in the dense ECM. Cells used microvilli to interact with each other: see as an example the green arrows in Figure 3C‐viii. Other types of cell–cell interaction were seen through longer and thicker projections between cells: see green arrow in Figure 3C‐vi. Interaction between the cells and the surrounding hydrogel was also seen through membrane projections that ended in the surface of the hydrogel, clearly suggesting that PANC‐1 cells actively interact with the EW/gelatin hydrogel and form cell–matrix unions (orange arrows in Figure 3C‐ix).

Abundant spherical particles (Figure 3C, yellow arrows) were seen in these MPS particularly at the boundaries between cells as well as on the cells surface. Some of these particles were larger than 1 µm in diameter (blue arrows in Figure 3C‐vii,viii). These particles could be EVs secreted by the PANC‐1 cells for intercellular communication. However, they could also be hydrogel globular particles grown in size by mineralization phenomena from the cell culture medium. Nevertheless, the typical nanoglobular morphology of the hydrogel could be clearly seen in gaps between cells (light orange asterisk in Figure 3C‐ix). In these gaps, we could also see individual rounded particles much larger in size (yellow arrows in Figure 3C‐ix), indicating that they may be EVs. Observation of the inner structure of the aggregates could discern whether some of these spherical particles are secreted by the cells and therefore, they could be distinguished as EVs.

**FIGURE 5:**
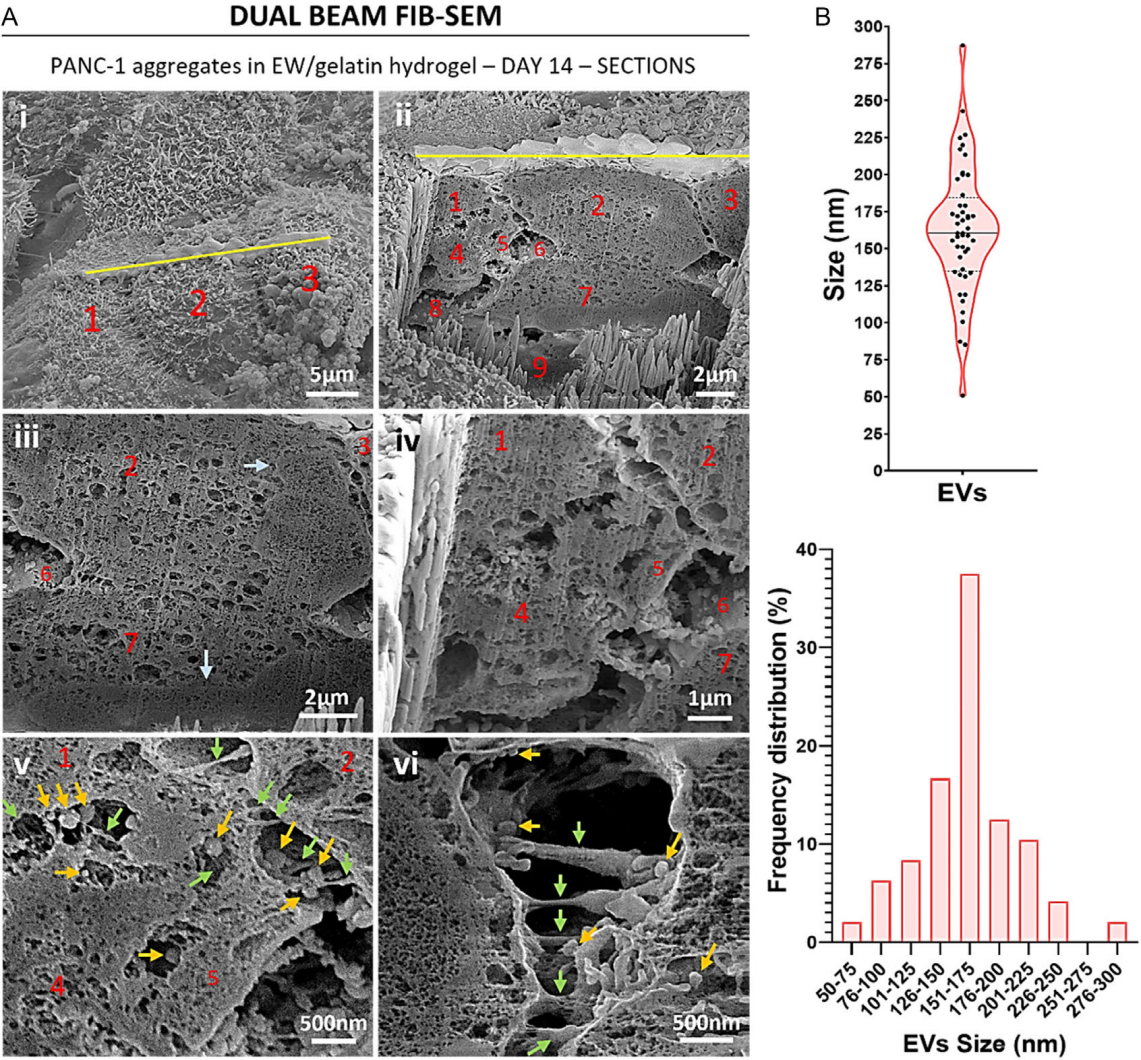
(A) Dual beam FIB‐SEM representative images of the internal cell organization of the aggregates formed by PANC‐1 cells in EW/gelatin hydrogels. Image i shows the surface of the aggregate milled with the ion beam along the yellow line. Images ii–v show internal sections of the aggregate shown in image i. Image vi belongs to a different aggregate. Individual cells are indicated by red numbers. For additional guidance to identify cell boundaries, Figure S1 provides the same images with a colored overlay mask highlighting the cellular regions. Light blue arrows: darker and denser intracellular areas. Green arrows: membrane projections connecting adjacent cells. Yellow arrows: EVs secreted by the cells. (B) Measurements of the size (i.e., diameter) of the EVs secreted by the PANC‐1 cells. Data shown in violin plot as its distribution with median and the IQR, each dot represents a measurement (*n *= 48).

Milling of the PANC‐1 aggregates revealed their internal organization (Figure 5A). In some cases, a gap was observed between the cells: see as an example the gap between cells 2 and 3 in Figure 5A‐ii. On the other hand, no gap was seen between some cells, which appeared fused: see as an example the absence of a gap between cells 2 and 7 in Figure 5A‐ii,iii. Where a gap was observed between cells, interaction between them was seen through membrane projections: see green arrows in Figure 5A‐v,vi. Darker, denser areas inside the cells could be seen, possibly indicating cell organelles: see light blue arrows in Figure 5A‐iii. Interestingly, spherical particles were seen in the gaps between cells (yellow arrows in Figure 5A‐v,vi) and many of them appeared associated to the membrane projections that connected adjacent cells (yellow arrows in Figure 5A‐v,vi). These spherical particles were most likely EVs secreted by the cells for intercellular communication. Measurement of the size, i.e. diameter, of the EVs secreted by the cells and seen after milling of the aggregates, showed that they were between 50 and 300 nm in size, with the majority of them in the 100–200 nm range (Figure 5B).

### 3.2 MPS of the TME of Lung Adenocarcinoma: A549 Cells in Collagen I Hydrogels

**FIGURE 6:**
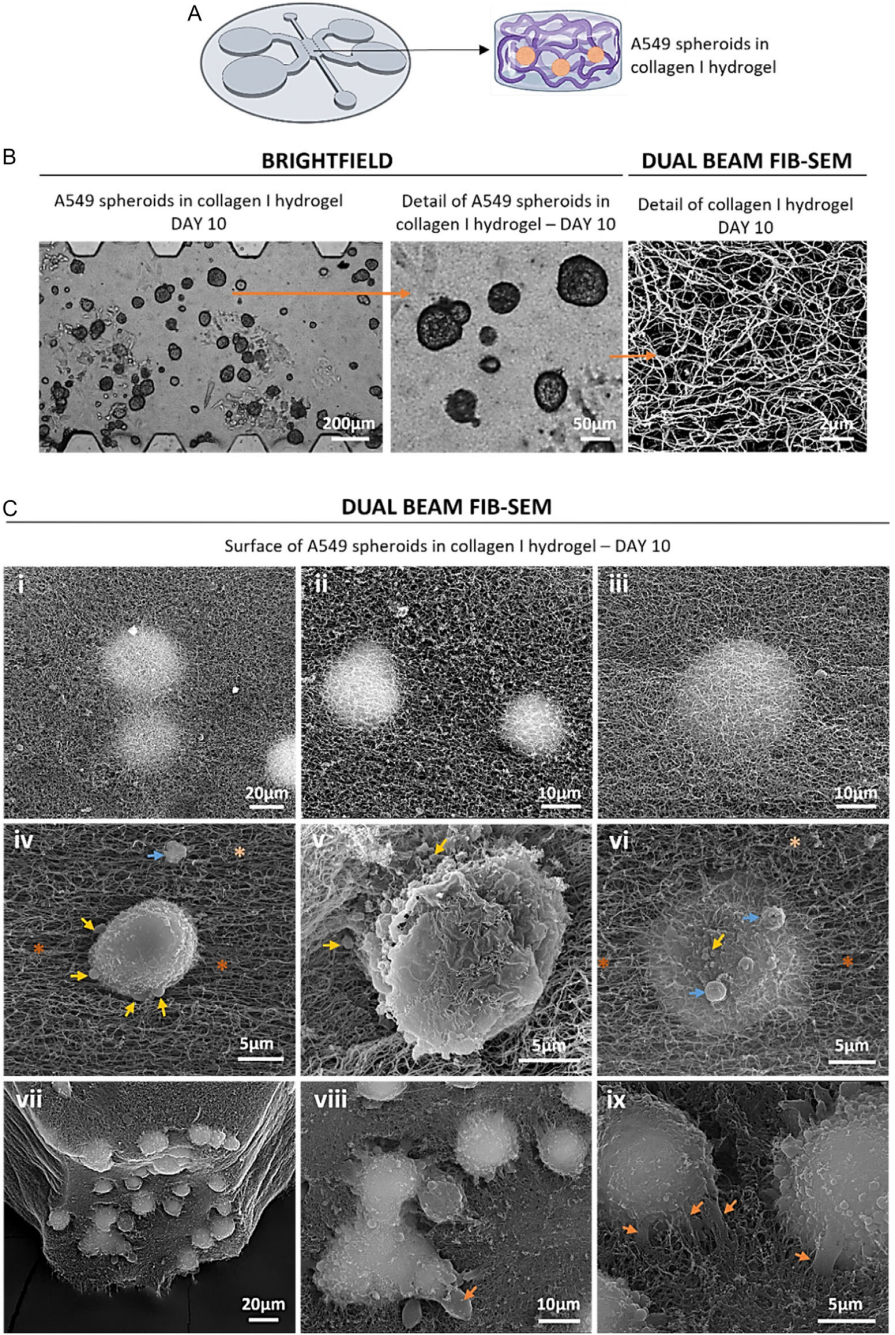
(A) Scheme of the MPS of the TME of LAC, consisting of a hydrogel‐based cancer‐on‐a‐chip model embedding A549 cells in collagen type I. (B) Brightfield microscopy representative images of the A549 spheroids grown over 10 days of culture in collagen I hydrogels. Zoomed area shows detail of spheroids. Dual beam FIB‐SEM representative image of collagen I hydrogel after 10 days of culture, displaying the typical nanofibrous morphology of these hydrogels. (C) Dual beam FIB‐SEM representative images of the surface of A549 spheroids in collagen I hydrogel. Dark orange asterisk: areas of aligned collagen fibers. Light orange asterisk: areas of randomly‐aligned collagen fibers. Orange arrows: unions between the cells and the hydrogel. Yellow arrows: spherical vesicles. Blue arrows: large spherical vesicles.

The collagen I hydrogels used in this study (6 mg/mL) were previously characterized by our group [14, 23, 24]. Rheological measurements indicate viscoelastic behavior and a reduced mesh size, consistent with low pore size and permeability of the matrix. These collagen hydrogels have a heterogeneous fibrous architecture with interconnected pores. When cells from the LAC cell line A549 were cultured in collagen type I hydrogels they formed small compact spheroids that under brightfield microscopy displayed a thick and dark outer layer and a clearer inner zone (Figure 6A,B). A previous study by our group showed that A549 spheroids grown in collagen I hydrogels are indeed small, with some of them measuring under 20 µm in diameter, and showing a very compact nature with tightly packed cell nuclei [21]. In this context, Figure S2 illustrates the typical growth kinetics of these spheroids, their metabolic activity over time, and representative structural changes observed upon exposure to a treatment, highlighting that these MPS have been successfully employed for functional studies of metabolic activity [21], growth [23], and treatment response [8, 25]. However, this study did not report on important ultrastructural features like cell–cell unions, cell–matrix interaction, or intercellular communication.

Observation under FIB‐SEM confirmed the compact nature of A549 spheroids which were completely embedded in the collagen I fibrous mesh: Figure 6C‐i–iii show the top side of spheroids that are underneath the collagen mesh, whilst Figure 6C‐vii shows the whole depth of the collagen gel and numerous spheroids through the fibrous gel. In terms of fine detail of external cell organization, unlike the PANC‐1 aggregates just described, the individual cells are not discernible, and a continuous external cell layer bounds the A549 spheroids (Figure 6C). Spherical vesicles are seen budding off the outer layer of the spheroids and eventually are secreted into the surrounding matrix: see yellow and blue arrows in Figure 6C‐iv–vi, and although not marked, can also be easily observed in images viii and ix. Some of these vesicles are visibly larger than 1 µm: see blue arrows in Figure 6C‐iv,vi. Easily visible too are the membrane projections that connect the spheroids with the collagen gel: see orange arrows in Figure 6C‐viii,ix. These membrane projections had a wide range of thicknesses and some of them were ameboid‐like (orange arrow in Figure 6C‐viii). Furthermore, we could also see that the spheroids seemed to align the fibers of the collagen hydrogel in one direction on opposite sides of the spheroid: see dark orange asterisks in Figure 6C‐iv,vi and compare with the random alignment of the original matrix (Figure 6B) pointed by the light orange asterisks in Figure 6C‐iv,vi.

**FIGURE 7:**
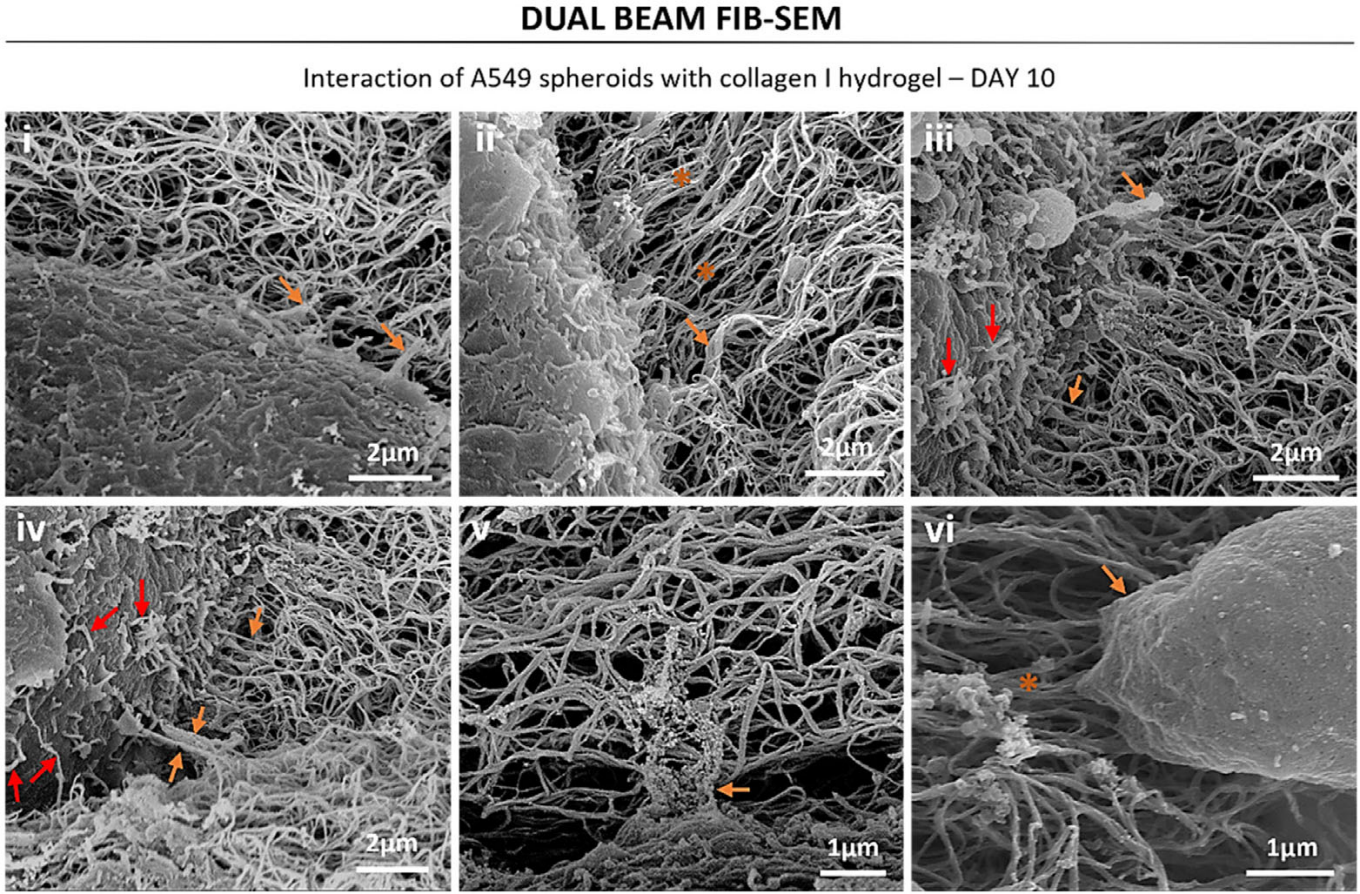
Dual beam FIB‐SEM representative images of interaction between A549 spheroids and the surrounding collagen I matrix. Dark orange asterisk: areas of aligned collagen fibers. Orange arrows: unions between the cells and the hydrogel. Red arrows: short fibers secreted by the spheroids.

Closer inspection of the cell–matrix interaction was carried out (Figure 7) confirming the variety of cell projections used to interact with the matrix (orange arrows in Figure 7). Figure 7‐vi shows an ameboid‐like projection interacting with the collagen matrix, and at the tip of it, we see aligned collagen fibers in the direction of the ameboid projection (dark orange asterisk). Furthermore, some of the projections seemed to be complexes with various elements involved, as seen in Figure 7‐v. Importantly, closer inspection of the spheroids surface showed evidence of some kind of fibers secretion: see red arrows in Figure 7‐iii,iv. However, it was difficult to clearly distinguish between secreted fibers and those belonging to the collagen matrix, and therefore, measurements of the secreted fibers were not attempted to avoid erroneous data.

**FIGURE 8:**
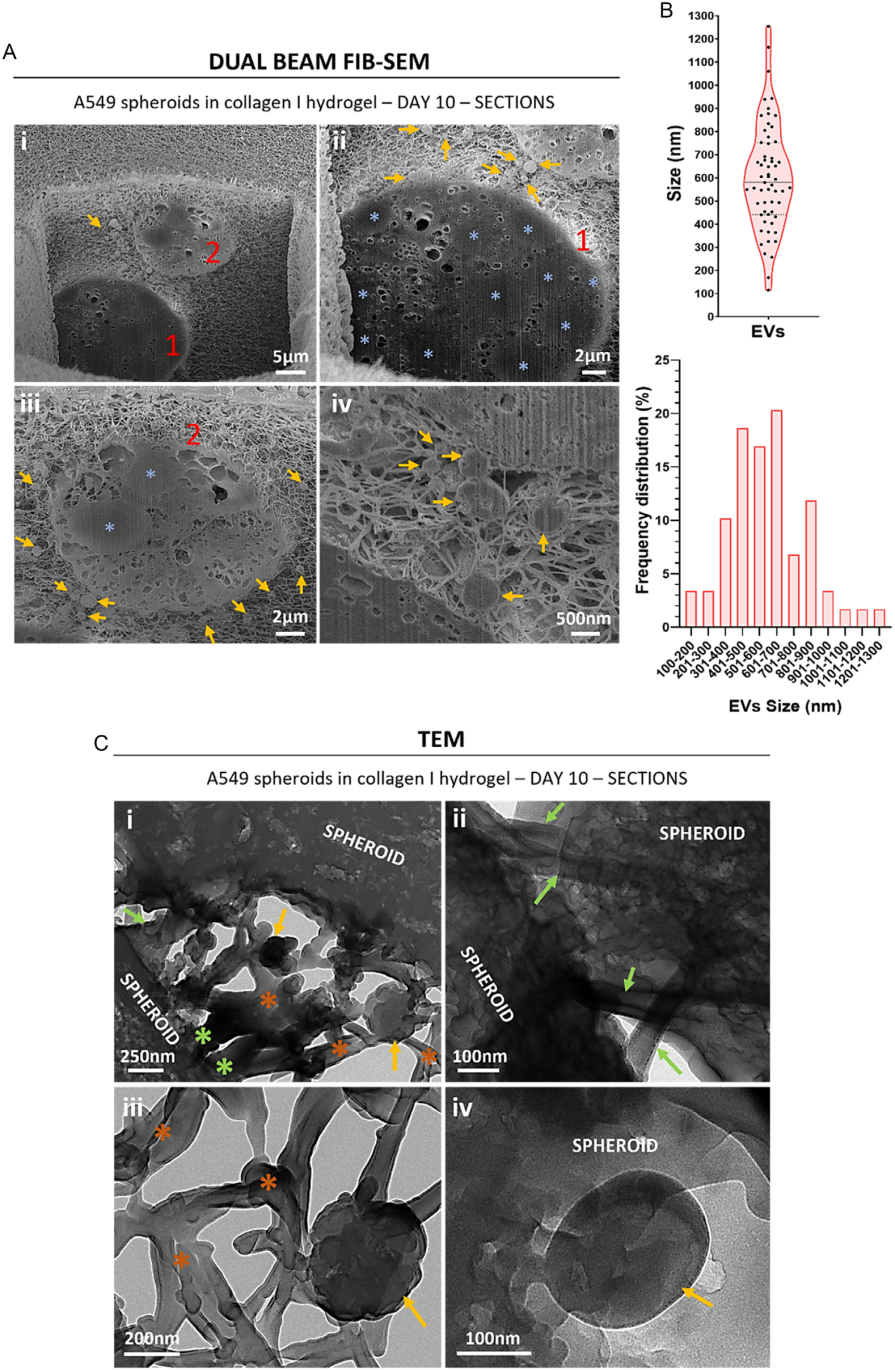
(A) Dual beam FIB‐SEM representative images of the internal cell organization of the spheroids formed by A549 cells in collagen I hydrogels. Image i shows two small spheroids embedded in collagen matrix. A different section of the two same spheroids was shown in reference [21]. Images ii and iii show each spheroid more in detail. Image iv belongs to two different spheroids from those in image i. Individual spheroids are indicated with red numbers. Light blue asterisks: cell nuclei. Yellow arrows: EVs secreted by the cells. (B) Measurements of the size (i.e., diameter) of the EVs secreted by A549 cells. Data shown in violin plot as its distribution with median and the IQR, each dot represent a measurement (*n *= 48). (C) TEM representative images of spheroids formed by A549 cells in collagen I hydrogels. Green asterisks: membrane projections into the collagen fibers. Orange asterisks: collagen fibers. Green arrows: unions between adjacent spheroids. Yellow arrows: EVs secreted by the cells.

Milling of the A549 spheroids revealed their internal organization (Figure 8A). Figure 8A‐i–iii shows two small spheroids completely embedded in the collagen matrix where tightly packed nuclei are seen as darker areas: see light blue asterisks in the mentioned images. Other than the individual cell nuclei, the boundary between adjacent cells cannot be distinguished, indicating a strong cell–cell contact. Clearly visible even at low magnification are the EVs secreted by the spheroids: see yellow arrows in Figure 8A. The EVs are entangled in the collagen matrix. Measurement of the size, i.e., diameter, of the EVs secreted by the cells and seen after milling of the spheroids, showed that they were between 100 and 1,250 nm in size, with the majority of them in the 300–900 nm range (Figure 8B).

Further ultrastructural observation of LAC‐on‐a‐chip samples was carried out by TEM, which allowed us to observe features not seen before with FIB‐SEM. Conventional TEM images appear clearer than our ones as they are usually mounted on a solid support. However, that was not the case for our lamellas, which were porous and let the light pass through, even creating shadows (Figure 8C). New ultrastructural features observed were unions between adjacent spheroids (green arrows in Figure 8C‐i,ii), which was hinted at by FIB‐SEM images like Figure 6C‐v,viii. Membrane projections into the collagen fibers are also observed: see green asterisks in Figure 8‐i. EVs are also observed associated to collagen fibers: yellow arrows in Figure 8C point to EVs, whilst orange asterisks mark collagen fibers. Finally, we observed an EV being released by a spheroid (Figure 8C‐iv).

Table 1 offers a summary of the ultrastructural features described for the two different models used as examples in this study, with the features observed for each MPS highlighted. This table provides a concise reference of the key structural characteristics captured using our workflow and illustrates the reproducibility and applicability of the methodology across different hydrogel‐based cancer‐on‐a‐chip systems.

**TABLE 1:** Summary of the ultrastructural features that were observed in this study for the two different MPS used as examples: PDAC‐on‐a‐chip and LAC‐on‐a‐chip.

Ultrastructural feature	PDAC‐on‐a‐chip: PANC‐1 cells in EW/gelatin hydrogels	LAC‐on‐a‐chip: A549 cells in collagen I hydrogels
External cell organization	‐ Large aggregates resembling grape‐like clusters. ‐ Compact aggregates with granular surfaces of stacked cells of 5–10 μm in diameter. ‐ Numerous microvilli observed.	‐ Small compact spheroids with a continuous external cell layer. ‐ Individual cells are not discernible. ‐ Microvilli not observed.
Internal cell organization	‐ Adjacent cells could either have a gap between them or appeared fused. ‐ Where a gap existed, membrane projections connected adjacent cells.	‐ Cells appear fused with tightly packed nuclei.
Cell–cell interactions	‐ Through microvilli and other longer and thicker projections.	‐ Strong cell–cell contact. ‐ Membrane projections connecting adjacent spheroids.
Cell–matrix interaction	‐ Through membrane projections ending in the hydrogel.	‐ Through membrane projections ending in the hydrogel. ‐ The membrane projections had a wide range of thicknesses and some of them were ameboid‐like.
Secretion of an ECM	‐ Structured dense matrix forming a coating over the cells surface. ‐ Formed by short fibers of 40–180 nm in diameter and 200–1,600 nm in length with random orientation.	‐ Short fibers secreted.
Intercellular communication through EVs	‐ Internally, seen in the gaps between cells, associated to membrane projections that connected adjacent cells. ‐ 50–300 nm in size.	‐ Spherical vesicles bud off the outer cell layer and are secreted into the surrounding matrix. ‐ The EVs are entangled in the collagen matrix. ‐ 100–1250 nm in size

## 4 Discussion

MPS of the TME have emerged as powerful tools in cancer research. In particular, cancer‐on‐a‐chip models incorporating 3D multicellular tumor structures and a hydrogel‐based matrix are highly biomimetic, as they incorporate important elements of the TME, such as complex cell‐to‐cell interactions, organization, O_2_ and pH gradients, and nutrients. The hydrogel matrix also acts as an ECM that the cells can interact with [5, 6, 10]. In this paper, we perform ultrastructural characterization of two previously developed cancer‐on‐a‐chip models by our group, therefore serving as examples [6, 21]. In our previous work, the choice of hydrogel composition was tailored to the specific cell type to best mimic the relevant TME and support optimal 3D growth. LAC cells were cultured in collagen hydrogels, reflecting the collagen‐rich stroma typical of lung tissue [8, 21]. In contrast, PDAC cells were embedded in a hybrid EW/gelatin hydrogel, mimicking the dense and stiff ECM characteristic of pancreatic tumors [6]. This hydrogel‐based approach enabled the development of well‐organized multicellular structures for each cell type and, importantly, provided a platform to investigate how both cell type and hydrogel composition influence structural organization within the cancer‐on‐a‐chip framework. The work presented in this paper establishes a versatile and reproducible methodological platform for ultrastructural characterization of 3D multicellular tumor structures in miniaturized hydrogel‐based models. While this study is primarily descriptive and methodological in nature, it demonstrates the feasibility and robustness of integrating advanced electron microscopy into MPS. Rather than replacing functional or fluorescence‐based analyses, the proposed approach complements existing techniques by providing nanoscale structural information that is otherwise inaccessible. As such, it offers a technical foundation for future systematic and comparative investigations of different hydrogels, cell types, and microenvironmental conditions in engineered tumor models.

The overall development of 3D multicellular tumor structures, whether in tissue culture plates or in MPS, is commonly monitored using optical microscopy techniques, e.g., brightfield microscopy, at designated time points, thereby generating images that are then used to quantify growth in size [6, 13]. For instance, as shown in our results, it is clearly observed from brightfield microscopy images that the 3D multicellular structures formed by PANC‐1 and A549 cells in their respective hydrogels are morphologically different, not only in terms of size and shape, but also in terms of cell organization (Figures 3B and 6B). Nevertheless, the limited resolution of optical microscopy leads to the use of other microscopy techniques for further imaging of 3D multicellular structures. It is the case of fluorescence, light‐sheet, or confocal microscopies, which may offer a higher degree of detail of these structures in terms of internal organization, expression of specific cellular markers or ECM structure [6, 21, 26].

While optical microscopy of MPS is usually straightforward, and fluorescence microscopy can be too if the cells are fluorescently labeled, confocal and light‐sheet microscopies require ample time for both sample preparation and observation. Regardless, these techniques do not reach sufficient degree of detail and important aspects that belong to the realm of ultrastructural characterization, remain unseen. These ultrastructural aspects include fine detail of cell organization, both external and internal, cell–matrix interaction, cell–cell unions, or intercellular communication via EVs. This minute and detailed degree of observation can only be accomplished using electron microscopy techniques. However, these techniques are rarely used in MPS mainly due to the difficulty that sample preparation for electron microscopy observation techniques entails, which is particularly aggravated in cancer‐on‐a‐chip models due to their miniaturized size. Hence, the motivation behind our work presented here.

For our study, we used FIB‐SEM over conventional SEM as it offers important advantages due to the addition of a FIB. FIB‐SEM allows for automated serial sectioning and imaging, enabling precise modification of samples [20, 27]. While SEM is excellent for surface imaging, FIB‐SEM enhances its capabilities by providing depth information and the ability to prepare samples for other techniques like TEM. Crucially for our study aim, FIB‐SEM uses a focused ion beam to mill away very thin layers of the sample after each SEM imaging pass [27]. This interesting feature of FIB‐SEM allowed us to observe the inner zone of the 3D multicellular tumor structures of our different MPS. Furthermore, we used FIB to prepare thin lamellas for TEM, allowing for higher‐resolution analysis of specific regions of interest [27]. In terms of potential artefacts during sample preparation and imaging, in the proposed workflow, sample shrinkage is minimized due to the physical confinement of the hydrogel‐based cultures within the microfluidic device architecture during fixation and processing. In addition, no resin infiltration is performed, thereby avoiding artefacts commonly associated with resin embedding and extraction. Nevertheless, care must be taken to ensure complete filling of the central chamber of the device during fixation, staining and dehydration steps to avoid local dehydration or sample collapse. Regarding FIB‐SEM imaging, we acknowledge that curtaining effects may occur during ion‐beam milling. In our experience, these effects can be mitigated by adjusting milling parameters and, when necessary, by performing additional cuts at adjacent locations to confirm the reproducibility of observed features. Charging artefacts were minimized through appropriate conductive coating and imaging conditions, as described in the Materials and Methods section.

In terms of external cell organization, our FIB‐SEM images revealed very different types of 3D multicellular structures in the two models used as examples. Both types of cells used in this study are derived from adenocarcinomas and both are epithelial, however, the 3D structures that they formed were strikingly different. Whilst PANC‐1 cells formed large grape‐like clusters with discernible individual cells, A549 cells formed small round and compact spheroids that appeared like a fusion of multiple cells. Another important morphological feature was in the abundance of microvilli observed in PANC‐1 aggregates. Microvilli have been found to be more abundant on cancer cells with high growth potential and metastatic ability compared to normal cells or cancer cells with low metastatic potential [28, 29]. This difference in microvilli presence is linked to the increased ability of cancer cells to exchange nutrients, adhere to other cells or tissues, and potentially resist immune responses [30]. Microvilli significantly increase the surface area of a cell, which can enhance the uptake of nutrients and the exchange of molecules with the surrounding environment. In PDAC, characterized by a dense desmoplastic stroma and hypomicrovascularity, basal microvilli are associated with higher glucose uptake, indicating a link between microvilli and the metabolic capacity of cancer cells [29]. Therefore, the numerous microvilli observed for our PANC‐1 aggregates might indicate a high metabolic activity, growth and metastatic potential, which correlates with the notorious aggressiveness of PDAC [29]. Interestingly, PANC‐1 cells on the surface of the aggregates were seen to use microvilli for cell–cell interaction, revealing a communication function for microvilli in PDAC cells. Further research could investigate the mechanism of microvilli communication in pancreatic cancer cells, perhaps opening a window for new therapeutic strategies. For A549 cells, studies have shown that the culture conditions and substrate influence the presence of microvilli [31, 32]. However, these studies were carried out in monolayer and our study provided a 3D environment to the A549 cells.

Cell–cell interactions were seen for PANC‐1 cells on the surface of the aggregates, which consisted of microvilli interaction and longer and thicker membrane projections. However, since the A549 spheroids had a continuous external layer, no cell–cell interactions were seen. Internally, we observed cell–cell interaction for PANC‐1 cells through membrane projections that connected adjacent cells with a gap between them. Otherwise, PANC‐1 cells would appear fused, same as A549 cells in the spheroids. Interestingly, interactions between A549 spheroids were hinted at by FIB‐SEM and revealed by TEM. These interactions between spheroids may be an initial step in their fusing to form a larger spheroid. Further research should investigate the families of proteins involved in the cell–cell and spheroid–spheroid interactions observed here.

The use of a nanoglobular hydrogel allowed us to observe matrix fibers production by PANC‐1 aggregates. These matrix fibers had diameters of 40–180 nm and lengths between 200 nm and 1.6 µm. The main fibrous proteins in the ECM of tissues are fibrillar collagens, elastins, fibronectins, vitronectin, fibrillins, and laminins [33]. Further research could identify the composition of the fibers produced by the PANC‐1 aggregates. Nevertheless, our observations demonstrate that pancreatic cancer cells produce matrix without the need to interact with other relevant cells of the TME like cancer‐associated fibroblasts (CAFs) or pancreatic stellate cells (PSCs), which have been described in the literature as major players in the production of the aberrant desmoplastic matrix of PDAC [34]. For the A549 cultures, we also observed production of matrix fibers, but it was not as clear as with the PANC‐1 cells due to the fibrous nature of the collagen I hydrogel used. What we did observe for the A549 cultures was how the spheroids remodel the surrounding collagen I matrix by aligning its fibers. Particularly important was the observation of ameboid‐like projections interacting with the collagen fibers, with aligned collagen fibers in the direction of the ameboid projection, indicating that matrix remodeling through mechanical alignment of the collagen fibers is necessary for cell migration, since ameboid cell projections are involved in cell locomotion [35]. Overall, ultrastructural observation of cell–matrix interaction and matrix production may help understanding how cancer cells modify their environment to promote tumor growth and spread. Coculture studies have already demonstrated that stromal, vascular, and immune cells significantly influence tumor behavior; therefore, future studies incorporating such coculture systems will be essential to determine how these additional components of the TME further modulate matrix remodeling, invasion‐related ultrastructure, and the associated biophysical properties through the ultrastructural analysis proposed in this work [6, 24, 36, 37].

One of the most important observations in this study was in the intercellular communication via EVs, which are membrane‐bound vesicles released by cells into the extracellular space. EVs act as a key mechanism for intercellular communication and cellular cargo transfer (e.g., proteins, lipids, nucleic acids) between cells, enabling signaling and regulatory pathways that influence cell function and behavior [38, 39, 40, 41]. EVs play a crucial role in pathological processes like cancer, where they have been shown to promote cancer cell proliferation, migration, invasion and metastasis [39, 42, 43]. EVs are heterogeneous in size, which is one of the criteria used to classify them into the different types, including exosomes (30–150 nm), microvesicles (100–1000 nm), and apoptotic bodies (1–5 µm) [38, 40]. Besides, cancer cells produce oncosomes, 100–400 nm vesicles carrying oncogenic macromolecules, and large oncosomes, which are 1–10 µm in size, contain oncogenic material and are cancer‐specific [39]. EVs size can be measured using several techniques, of which electron microscopy techniques like SEM or TEM are considered the gold standard [38]. Their main disadvantage is the time that it takes to prepare the sample for observation [38]. In this study, we used the section SEM images generated by FIB to quantify the size of the visible EVs secreted by the cells. EVs in the A549 cultures were visibly larger than those observed in the PANC‐1 cultures in EW/Gel hydrogels, suggesting that A549 cells use different types of EVs for communicating than PANC‐1 cells. Based on size, we speculate that PANC‐1 cells mostly release exosomes together with oncosomes and small microvesicles, while A549 cells may generate a mix of exosomes, microvesicles, oncosomes, and even large oncosomes, since there is evidence in the literature of secretion of large oncosomes by A549 cells [44]. Further research could characterize the cargo of these different EVs, presumably directed towards encouraging cancer cell proliferation and invasion [43]. An important observation was that in the PANC‐1 cultures, the EVs were associated to the cell–cell unions, suggesting that EVs use cell unions for target delivery of cargo in pancreatic cancer cells. Therefore, it may be speculated that by disrupting these cell–cell unions the intercellular communication via EVs of pancreatic cancer cells would also be disrupted. In the A549 cultures, EVs were seen emerging from the outer spheroid membrane, and released into the immediate collagen matrix, where we observed them associated to the collagen fibers, suggesting that the EVs produced by A549 are capable of interacting with collagen fibers, which has been previously suggested by other researchers. For instance, using electron microscopy techniques and a pull‐down assay, Palmulli and colleagues showed that melanoma cells release diverse subpopulations of small EVs that differentially interact with collagen fibers [45]. The interaction of EVs with collagen supports the suggested role of EVs in cancer matrix remodeling, thereby contributing towards cancer progression, as well as their also suggested role in regulating the directional migration of tumor cells [45, 46]. Our ultrastructural observations suggest that MPS encompassing cancer‐on‐a‐chip models are important systems for the study of intercellular communication via EVs between cancer cells.

## 5 Conclusions

Decades of research have shown the importance of studying ultrastructural features to understand pathological processes or inform disease diagnosis. Yet, ultrastructural studies in the promising cancer‐on‐a‐chip models are practically nonexistent due to the complexity of sample preparation for electron microscopy techniques, which is particularly aggravated with these miniaturized models. Our results show that following our sample preparation technique it was possible to observe the external and internal organization of 3D multicellular tumor structures in close detail, cell–matrix unions, cell–cell unions, spheroid–spheroid unions, matrix production, and intercellular communication via EVs. We believe that this study demonstrates the feasibility of using advanced electron microscopy techniques to observe ultrastructural features of miniaturized cancer models, revealing a new dimension, i.e., ultrastructure, in the use of these models to study tumor processes and find new therapeutic targets.

## Supporting Information

Additional supporting information can be found online in the Supporting Information section.

## Author Contributions

**Paula Guerrero‐López:** conceptualization (equal), data curation (equal), formal analysis (equal), funding acquisition (equal), investigation (equal), methodology (equal), writing – original draft (lead), writing – review & editing (equal). **Karinna Georgiana Pele:** investigation (equal), methodology (equal), writing – review & editing (equal). **Mariano Barrado:** investigation (equal), methodology (equal), writing – review & editing (equal). **Pilar Alamán‐Díez:** investigation (equal), methodology (equal), supervision (equal), writing – review & editing (equal). **José Manuel García‐Aznar:** funding acquisition (equal), project administration (equal), resources (equal), supervision (equal), writing – review & editing (equal). **Elena Garcia‐Gareta:** conceptualization (equal), data curation (equal), formal analysis (equal), project administration (lead), resources (lead), supervision (equal), writing – original draft (supporting), writing – review & editing (equal).

## Funding

This study was supported by the European Research Council (ERC) under the European Union's Horizon 2020 research and innovation programme (ICoMICS grant agreement No 101018587). E.G‐G gratefully acknowledges her “Ramon & Cajal Fellowship” (RYC2021–033490‐I, funded by MCIN/AEI/10.13039/501100011033 and the EU “NextGenerationEU/PRTR”). Dual‐beam FIB‐SEM characterization was funded by ICTS ELECMI grants ELC217–2023 and ELC384–2024. KG.P gratefully acknowledges the Grants Program for Master students at Aragon Institute of Engineering Research (I3A). P.G‐L gratefully acknowledges the Department of Science, University, and Knowledge Society of the Government of Aragon (predoctoral contract, Call No. 2021–25).

## Conflicts of Interest

The authors declare no conflicts of interest.

## Supporting information

Supplementary Material

## Data Availability

The data that support the findings of this study are available from the corresponding author upon reasonable request.
