# Supplementary material for: Ultrastructural Study of Microphysiological Systems of the Tumor Microenvironment
Source: Small Sci. 2026 Apr 24;6(4):e202500567. doi: 10.1002/smsc.202500567 (PMC13116338; doi:10.1002/smsc.202500567)
Supplement: Supplementary file 1 — Supplementary Material [file SMSC-6-e202500567-s001.pdf]

# Supplementary Information

## Ultrastructural study of microphysiological systems of the tumour microenvironment

**Paula Guerrero-López[1], Karinna Georgiana Pele[1], Mariano Barrado[2], Pilar Alamán-Díez[1], José Manuel García-Aznar[1,3], Elena García-Gareta[1,3,4*]**

[1]Multiscale in Mechanical & Biological Engineering Research Group, Aragon Institute of Engineering Research (I3A), School of Engineering & Architecture, University of Zaragoza, 50018 Zaragoza, Aragon, Spain.
[2]Advanced Microscopy Laboratory, LMA-University of Zaragoza, 50018 Zaragoza, Aragon, Spain.
[3]Aragon Institute for Health Research (IIS Aragon), Miguel Servet University Hospital, 50009 Zaragoza, Aragon, Spain.
[4]Division of Biomaterials & Tissue Engineering, UCL Eastman Dental Institute, University College London, London NW3 2QG, United Kingdom.

* Corresponding author:
Elena García-Gareta
Aragon Institute of Engineering Research (I3A)
School of Engineering & Architecture
University of Zaragoza
Zaragoza, Aragon, Spain.
garciage@unizar.es

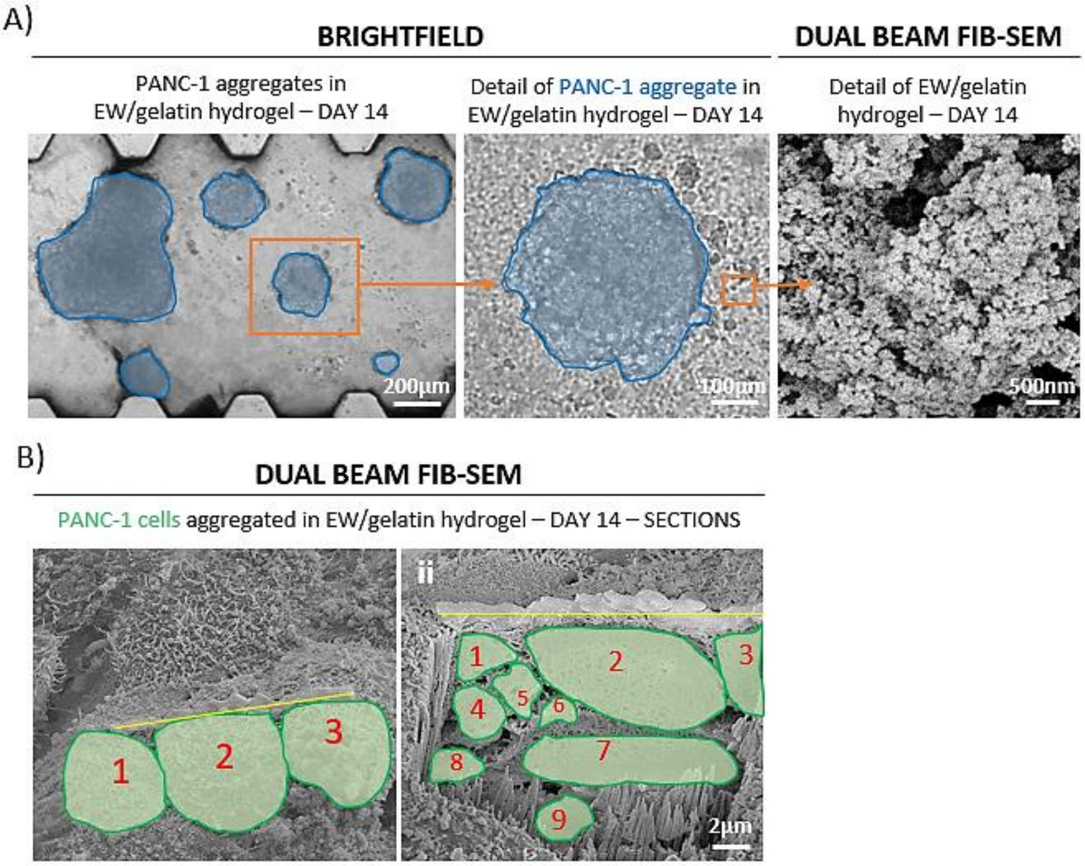


**Supplementary Figure 1. Visual guide for identifying cellular structures across imaging modalities.** A) Representative bright-field (BF) image showing multicellular tumor aggregates within the hydrogel. The aggregate is outlined in blue to assist in identifying cellular clusters in optical images. B) Representative electron microscopy (EM) image showing individual cells within the hydrogel matrix. Cellular regions are highlighted in green to facilitate the recognition of cells in grayscale EM images.

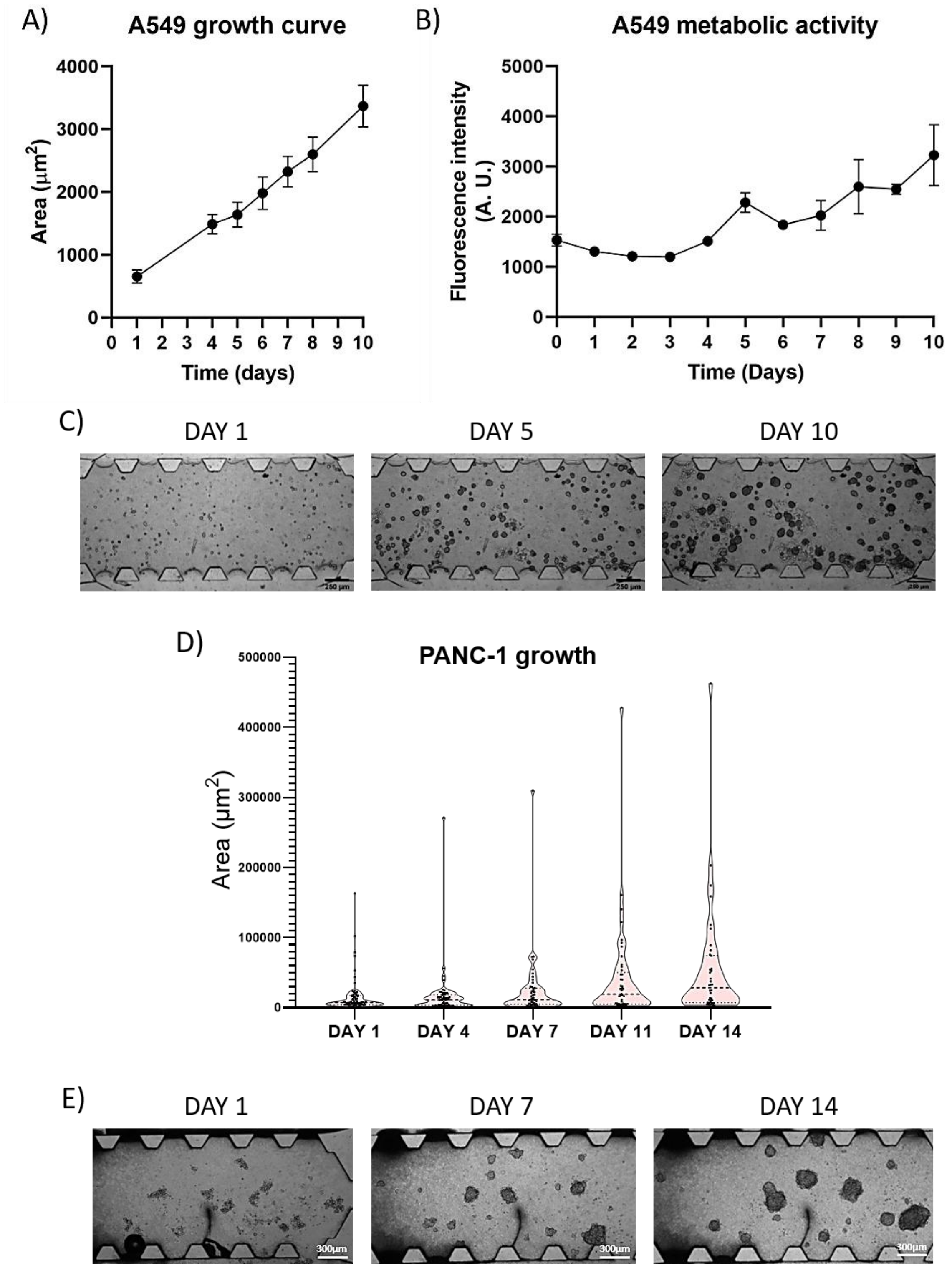


**Supplementary Figure 2. Biological characterization and ultrastructural comparison of 3D multicellular tumor structures.** A) Spheroid growth curve over a 10-day culture period for A549 cells cultured in a collagen I hydrogel. Data presented as mean ± SEM. B) Metabolic activity assessed by Alamar Blue over the same 10-day period. Data presented as mean ± SEM. C) Representative brightfield images showing visual growth of A549 spheroids in collagen I hydrogel over a 10-day culture period. Scale bar = 250µm. D) Growth of PANC-1 aggregates in EW/gelatin hydrogels over a 14-day culture period.

Data presented as violin plots showing median, interquartile range and individual data points. E) Representative brightfield images showing visual growth of PANC-1 aggregates in EW/gelatin hydrogel over a 14-day culture period. Scale bar = 300µm.
